# Fostering trust, collaboration, and a culture of continuous quality improvement: A call for transparency in medical school accreditation

**DOI:** 10.36834/cmej.70061

**Published:** 2020-09-23

**Authors:** Arshia Pedram Javidan, Lucshman Raveendran, Yeshith Rai, Sean Tackett, Kulamakan Mahan Kulasegaram, Cynthia Whitehead, Jay Rosenfield, Patricia Houston

**Affiliations:** 1 Faculty of Medicine, University of Toronto, Ontario,; 2 Institute of Health Policy, Management, and Evaluation, Dalla Lana School of Public Health, University of Toronto, Ontario, Canada; 3 Division of General Internal Medicine, Johns Hopkins Bayview Medical Center, MD, USA; 4 The Wilson Centre for Research in Education, University Health Network, University of Toronto, Ontario, Canada; 5 Women’s College Hospital, Ontario, Canada; 6 Department of Family and Community Medicine, Faculty of Medicine, University of Toronto, Ontario, Canada; 7 Schulich School of Medicine & Dentistry, Western University, Ontario, Canada; 8 Department of Anesthesia, Faculty of Medicine, University of Toronto, Ontario, Canada; 9 Department of Anesthesia, St. Michael’s Hospital, Unity Health, Ontario, Canada

## Abstract

Medical schools provide the foundation for a physician’s growth and lifelong learning. They also require a large share of government resources. As such, they should seek opportunities to maintain trust from the public, their students, faculty, universities, regulatory colleges, and each other. The accreditation of medical schools attempts to assure stakeholders that the educational process conforms to appropriate standards and thus can be trusted. However, accreditation processes are poorly understood and the basis for accrediting authorities’ decisions are often opaque.

We propose that increasing transparency in accreditation could enhance trust in the institutions that produce society’s physicians. While public reporting of accreditation results has been established in other jurisdictions, such as Australia and the United Kingdom, North American accrediting bodies have not yet embraced this more transparent approach. Public reporting can enhance public trust and engagement, hold medical schools accountable for continuous quality improvement, and can catalyze a culture of collaboration within the broader medical education ecosystem. Inviting patients and the public to peer into one of the most formative and fundamental parts of their physicians’ professional training is a powerful tool for stakeholder and public engagement that the North American medical education community at large has yet to use.

## Abstract (fr)

Les facultés de médecine procurent les bases pour la croissance professionnelle et le développement professionnel continu. Elles absorbent également une grande part des ressources gouvernementales. Conséquemment, elles devraient chercher des occasions de maintenir la confiance du public, de leurs étudiants, du corps professoral, des universités, des organismes de réglementation et les unes des autres. L’accréditation des facultés de médecine vise à assurer les parties prenantes que le processus éducationnel est conforme aux normes appropriées et donc de confiance. Toutefois, les processus d’accréditation sont mal compris et les fondements des décisions d’accréditation des autorités sont souvent opaques.

Nous proposons que l’accroissement de la transparence du processus d’agrément puisse rehausser la confiance dans les institutions qui forment les médecins de notre société. Bien que la diffusion publique des résultats de l’agrémentsoit établie dans d’autres juridictions, comme en Australie et au Royaume-Uni, les organismes d’agrément de l’Amérique du Nord n’ont pas encore adopté cette approche plus transparente. Les la diffusion publique peut améliorer la confiance et la participation du public, tenir les facultés responsables de l’amélioration continue de la qualité et catalyser une culture de collaboration au sein de l’écosystème élargi de la formation médicale. Inviter les patients et le public à scruter l’une des étapes les plus formative fondamentale de la formation professionnelle de leurs médecins est un puissant outil pour les parties prenantes ainsi que pour susciter la participation du public. Il reste à l’utiliser dans la communauté d’éducation médicale nord-américaine.

## Introduction

In Canada, medical schools undergo an eight-year accreditation cycle by the Committee on Accreditation of Canadian Medical Schools (CACMS) and the Liaison Committee on Medical Education (LCME).^1^ In the United States, this process occurs exclusively through the LCME.^2^ A mandatory part of accreditation is the Independent Student Analysis (ISA), a student-led survey that captures student opinions about nearly every aspect of their educational experience.^1^ The student authors of this commentary who led the ISA at their medical school for its most recent accreditation cycle (AJ, LR, YR) came to appreciate the quality and quantity of data generated and were disheartened to see this information constrained to the exclusive use by accreditation bodies and medical schools. While the ISA is a significant undertaking in itself, it is a small part of all the accreditation data collected. If accreditation data were publicly available, we argue it could accelerate continuous quality improvement (CQI) in undergraduate medical education (UME), foster public trust and engagement, and improve the educational experience for students and faculty alike.

## Current accreditation practices

CACMS and the LCME state that they will “disclose to the public only the accreditation status of the school” and that “the visit/survey report … transmitting the accreditation decision will be held confidential by CACMS/the LCME” with the option for schools to disclose the final report at their discretion.^3^^,^^4^ With the decision lying with individual institutions to publish data, significant differences exist among medical schools in the degree of transparency that they choose to enact. For example, in 2011, the University of Toronto was the first Canadian medical school to release to the public the results of the ISA as well as the full accreditation report and follow-up reports.^5^ They did this again for the 2019 cycle (the entirety of the 2019 ISA report and the Faculty’s response are available online).^6^ This decision was made by the school’s student and faculty leadership to promote a culture of transparency in accreditation amongst medical schools and to provide public accountability for the recommendations proposed within the report. Our search of the websites of each of Canada’s seventeen medical schools (including both a hand search as well as the search terms ‘accreditation,’ ‘independent student analysis,’ ‘self-study,’ as well as their combinations and variants in French) reveals significant variation in medical schools’ transparency with accreditation findings, with over half (Table 1, 10/17, 59%) having published none of their accreditation documents, and only five schools having published the final accreditation site team report. While the CACMS and LCME websites post a compilation of all currently accredited Canadian and allopathic American medical schools, there is no framework in place for the reporting of more detailed accreditation data.^7^^,^^8^

**Table 1:** Transparency of accreditation data for Canada’s 17 medical schools

	Accreditation data or results publicly available?			
Canadian Medical School/University Affiliation	Independent Student Analysis	Self-study report	Accreditation site team report	Final accreditation letter
University of Calgary	Yes^9^	Yes^10^	Yes^10^	No
Dalhousie University	No	No	Yes^11^	Yes^11^
McGill University	No	No	Yes^12^	Yes^12^
McMaster University	No	No	No	No
Memorial University of Newfoundland	No^13^^–^^15^	No^13^^–^^15^	No^13^^–^^15^	No^13^^–^^15^
Queen’s University	No	No	No	No
University of Manitoba	No	No	No	Yes^16^
University of Ottawa	Yes. ISA available with a confidentiality clause^17^	No	No	Yes^18^
University of Saskatchewan	No^19^	No^19^	No^19^	No^19^
University of Toronto	Yes^5,6^	Yes^5^	Yes^5^	Yes^5^
University of British Columbia	No^20^	No^20^	No^20^	No^20^
Western University	No^21^	No^21^	No^21^	No^21^
University of Alberta	No	No	Yes^22^ Interim accreditation report available	No
Université Laval	No	No	No	No
Université de Sherbrooke	No	No	No	No
Université de Montréal	No	No	No	No
Northern School of Medicine	No^23^	No^23^	N/A (currently undergoing)^23^	No^23^

The World Federation of Medical Education (WFME) states that the decisions on accreditation of medical programs must be made public and that the publication of the reports providing the basis for the decisions should also be considered for public view.^24^ The United Kingdom (UK) and Australia are two jurisdictions that have opted to publicly release detailed summaries of medical school accreditation results.^25^^,^^26^Findings from their accreditation processes are plain-language descriptions of the strengths, weaknesses, and recommendations for each medical school, with supporting data.^25^^,^^26^ The UK and Australian models provide a standard to strive for in terms of public reporting and transparency. To our knowledge, the effects of transparency of these accreditation findings on public confidence have not been studied. However, it seems reasonable to infer that public reporting of accreditation results would allow stakeholders to view accreditation data which may have previously been inaccessible. In turn, they can evaluate whether medical schools are functioning within their interests, which may provide public assurance in an effective accreditation process.

## Potential benefits of transparency

### Enhancing public trust and engagement

While many advocate for greater patient involvement in medical education, the information asymmetry between medical schools’ and the public’s knowledge of medical education imposes a significant barrier to patient involvement.^27^ Accreditation provides an ideal checkpoint to reorient the public to the quality and rigor of a medical school’s education program. Transparency and information sharing in this way yield a potential benefit of enabling and encouraging public engagement. Overall, the effects of transparency on public engagement have yet to be studied in the literature, and this is an area of further research. However, the UK and Australian reports indicate that sharing accreditation data can reveal the nature and extent of public involvement in training medical students and in curriculum development and can highlight further opportunities for public consultations.^25^^,^^26^

### Fostering collaboration

The accreditation process is intended to promote CQI within UME, similar to the CQI model emphasized in post-graduate medicine accreditation by the Royal College of Physicians and Surgeons of Canada and the College of Family Physicians of Canada.^28^ Although each medical school may face its unique challenges, many of the competencies and values that medical schools aim to promote are common across institutions, and medical schools could learn from the experiences of colleagues at other institutions through their accreditation data.^29^ Public release of accreditation results can foster a culture of openness, such that the release of results need not be perceived as ‘airing one’s dirty laundry’. Instead, through the acknowledgement of a school’s strengths and weaknesses, inter-institutional collaboration has the potential to fuel quality improvement, introduce financial efficiencies through economies of scale (e.g., schools may reduce costs associated with adding a new component to the MD program by identifying and avoiding inefficiencies when another program did the same), and allow schools to hold each other more accountable.^29^ We believe that while many forums exist for promoting collaboration, the potential for accreditation to foster collaboration has not yet been fully captured.

### Benefiting educators and researchers

Some educators may have limited understanding of accreditation and may view it as an administrative burden that inhibits innovation instead of promoting it.^30^ Making the results of accreditation transparent could raise awareness among educators about its value and catalyze ideas for improving the accreditation process itself. More broadly, as per the WFME, increasing access to accreditation data may improve international consistency in the quality of medical education and better guide quality improvement initiatives.^31^ We propose that one mechanism by which this may also occur is that it may broaden the pool of accreditation researchers who could access accreditation data to advance the limited evidence base for UME accreditation and improve accreditation’s return on investment for schools.

Additionally, individual clinician-educators may only have insight into their own particular facet of the medical curricula, with insufficient knowledge on how their role fits within the broader context of UME. This issue may be particularly salient for community-based clinical teaching faculty, where geographic separation from core teaching facilities and curriculum improvement processes may exacerbate the feeling of disengagement. Transparency in accreditation results may allow educators to have a more grounded sense of their contributions and provides the ability to engage in informed advocacy to meet accreditation recommendations and improve medical education.

### Overcoming reductionism of medical school rankings

Current systems of medical school rankings are based on metrics that do not necessarily reflect the quality of UME yet significantly influence public perception. For example, Prime Minister Trudeau tweeted the ranking of the University of Toronto Faculty of Medicine, which was subsequently widely disseminated.^32^ While in 2019 the University of Toronto Faculty of Medicine was ranked globally as the fifth best school for Clinical Medicine by *U.S. News & World* Report,^33^ these rankings are deeply biased as they do not consider the quality of UME, student engagement and satisfaction, the quality of clinical care provided by faculty and graduates, and ignore the value of medical school accreditation.^34^^,^^35^ Instead, these metrics focus on research productivity, student selectivity (undergraduate grades, standardized test scores, acceptance rate), and an internal self-assessment score.^34^^–^^37^ Although these rankings fundamentally misrepresent the mission of medical schools, they nevertheless have a substantial influence on public perception. Beyond this, the current model of ranking systems may encourage the use of financial capital to inflate rankings by medical schools with no direct improvement of educational quality.^38^

By embracing transparency in accreditation, medical schools and their accrediting bodies can be at the forefront of a fundamental paradigm shift in public perception. Publicly available accreditation reports would provide a detailed and more accurate description of metrics that truly reflect the missions of medical schools. These data could be used as a means of producing meaningful insights about medical schools for the public. Instead of reducing a medical school’s performance into a ranking derived from criteria not fully representing the quality of medical education, a school’s strengths, areas of improvement, and unique characteristics could be highlighted. This shift would appropriately move the conversation away from commercial third parties that currently, and often inaccurately, dominate the conversation, and provide public accountability for medical schools to further invest in their missions for society.^34^^,^^35^

## Moving past the limitations

Transparency may have certain drawbacks. It may be argued that increased transparency in accreditation processes may result in public interference with internal decision-making. For example, schools may worry that public knowledge of their challenges may affect future students’ decisions to apply. In evaluating this argument, we re-iterate that the effects of increasing transparency in medical school accreditation results have yet to be studied. However, examining transparency in accreditation in similar domains provides some context and suggests that public interference is unlikely. Public release of hospital accreditation results has been associated with a negligible impact on public decision-making and perceptions.^39^^,^^40^ Instead, there is evidence to suggest that public reporting of hospital accreditation results may be associated with increased leadership involvement in performance improvement, with a greater emphasis on improved outcomes in the hospital setting.^39^^,^^40^ Additionally, the World Health Organization in collaboration with the WFME suggests that transparency in accreditation can allow for more informed advocacy by leaders and educators.^24^ Collectively, the improved leadership capacity and additional accountability can work to accelerate quality improvement in medical education.

Another unintended consequence of increased transparency of accreditation findings may be that educators and medical schools become concerned about being perceived as inferior by their colleagues and the public.^30^ There may be a belief that accreditation data will impact their ability to recruit quality staff, will attract attention from other regulators, and potentially have a negative financial impact. However, we believe these hesitations may derive from the present culture of competition between medical institutions and that these consequences may become less concerning with a cultural shift. We envision that if transparency was present at a systemic level between medical institutions, a culture shift from competition to collaboration may allow educators to be more forthcoming and comfortable with their school’s weaknesses. Instead of boasting a passing grade or floundering in a failing grade, providing a standardized public record of the precise strengths and weaknesses of schools in both cases may actually reduce stigma and reframe the situation in a solutions-oriented way.

Our call to action is for a collective discussion on and inquiry into how accreditation can become more transparent to its stakeholders. Should North American medical schools and accrediting bodies choose to embrace transparency in reporting accreditation data, a number of benefits may be realized. These include paving the path for increased public trust, fueling quality improvement and collaboration between schools, providing direct benefits to educators and researchers, and potentially shifting the paradigm on public evaluation of medical schools. At the same time, the possible negative consequences and associated hesitations must also be kept in mind. We believe these can be mitigated through careful implementation, involving an open dialogue between medical schools and accrediting bodies, yielding a net benefit to schools and the public.
